# Supplementary material for: Hyaluronic Acid-Conjugated with Hyperbranched Chlorin e6 Using Disulfide Linkage and Its Nanophotosensitizer for Enhanced Photodynamic Therapy of Cancer Cells
Source: Materials (Basel). 2019 Sep 21;12(19):3080. doi: 10.3390/ma12193080 (PMC6803876; doi:10.3390/ma12193080)
Supplement: Supplementary file 1 [file materials-12-03080-s001.pdf]

Experimental

Materials

Methoxy poly(ethylene glycol)-amine (MePEG-amine) with molecular weight of 2,000 g/mol and 5,000 g/mol (MePEG amine 2K, MePEG-amine 5K) was purchased from Sunbio Co. Ltd. (Seoul, Korea).

^{1}H NMR spectra

Synthesis of Ce6 tetramer and Ce6 decamer conjugates was confirmed with ^{1}H NMR spectra (500 mHz superconducting Fourier transform (FT)-NMR spectrometer, Varian Unity Inova 500 MHz NB High Resolution FT NMR; Varian Inc, Santa Clara, CA).

Ce6tetra-MePEG 2K conjugates

27.5 mg of Ce6 tetramer was dissolved in 10 ml DMSO with EDAC (1.91 mg, 0.01 mM) and NHS (1.15 mg, 0.01 mM). This solution was magnetically stirred for 9h and then mixed with 20 mg MePEG-amine 2K in 5 ml DMSO. This solution was stirred for 36 h and then put into dialysis membrane (MWCO: 8,000 g/mol) to dialyze against water over 2 days. Following this, resulting solution was lyophilized for 3 days to obtain Ce6tetra-MePEG 2K conjugates as a solid. This solid was stored in refrigerator at -20 ^{o}C. Yield was approximately 98.6% (w/w) from mass measurement: Yield = [Weight of final product/(Weight of Ce6 tetramer + weight of MePEG-amine 2K)] × 100.

Ce6deca-MePEG 5K conjugates

Ce6 decamer (70 mg) was dissolved in 15 ml DMSO with EDAC (1.91 mg, 0.01 mM) and NHS (1.15 mg, 0.01 mM). This solution was stirred magnetically for 9h and then

mixed with 50 mg MePEG amine (M.W. = 5,000 g/mol) in 5 ml DMSO. This solution was further stirred for 36h and then put into dialysis membrane (MWCO: 8,000 g/mol). This was dialyzed against water over 2 days and lyophilized for 3 days to obtain Ce6deca-MePEG 5K conjugates as a solid. Final product was used to analyse or stored in refrigerator at -20 ^{o}C. Yield was approximately 99.2 % (w/w) from mass measurement: Yield = [Weight of final product/(Weight of Ce6 decamer + weight of MePEG amine 5K)] × 100.

Results

Figure S1(a) shows the ^{1}H NMR spectra of Ce6 tetramer. As shown in Figure S1(a), specific peaks of Ce6 and cystamine was confirmed at 1.0 ppm, 6.0 ~ 7.0 ppm and 2.8 ~ 3.2 ppm, respectively. These results indicated that Ce6 tetramer could be synthesized.

Figure S1(b) shows the ^{1}H NMR spectra of Ce6 decamer. As shown in Figure S1(b), specific peaks of Ce6 and cystamine was confirmed at 1.0 ppm, 6.0 ~ 7.0 ppm and 2.8 ~ 3.2 ppm, respectively. These results indicated that Ce6 decamer could be also synthesized.

Furthermore, Ce6 tetramer and Ce6 decamer was conjugated with MePEG-amine 2K and MePEG-amine 5K to characterize the hyperbranched Ce6 as shown in Figure S2. As shown in Figure S2(a) and (b), ethylene proton of MePEG and methylene proton of Ce6 tetramer or Ce6 decamer was confirmed at 3.6 ppm and 1.6 ppm, respectively, and the number of Ce6 in hyperbranched Ce6 was estimated from these peaks. As abbreviated in Table S1, the estimated number of Ce6 in hyperbranched Ce6 was 3.64 and 9.1, respectively. Even though the experimental number of Ce6 in Ce6 tetramer and Ce6 decamer was slightly lower than theoretical value, Ce6 tetramer and Ce6 decamer was successfully synthesized.

Figure S3 shows the emission spectra of Ce6. Ce6 showed maximum peak between 650 and 700 nm as similar to Ce6tetraHA or Ce6decaHA. Furthermore, the fluorescence spectra of Ce6 tetramer and Ce6 decamer were compared with Ce6 as shown in Figure S4. As shown in Figure S4, the fluorescence spectra of Ce6 tetramer and Ce6 decamer were not significantly different to the fluorescence spectra of Ce6.

Table S1. Characterization of Ce6tetra-MePEG 2K conjugated and Ce6decaMePEG 5K conjugates

	The number of Ce6 in hyperbranched Ce6	
	Theoretical	Experimental
Ce6tetraMePEG 2K conjugates	4	3.64
Ce6decaMePEG 5K conjugates	10	9.1

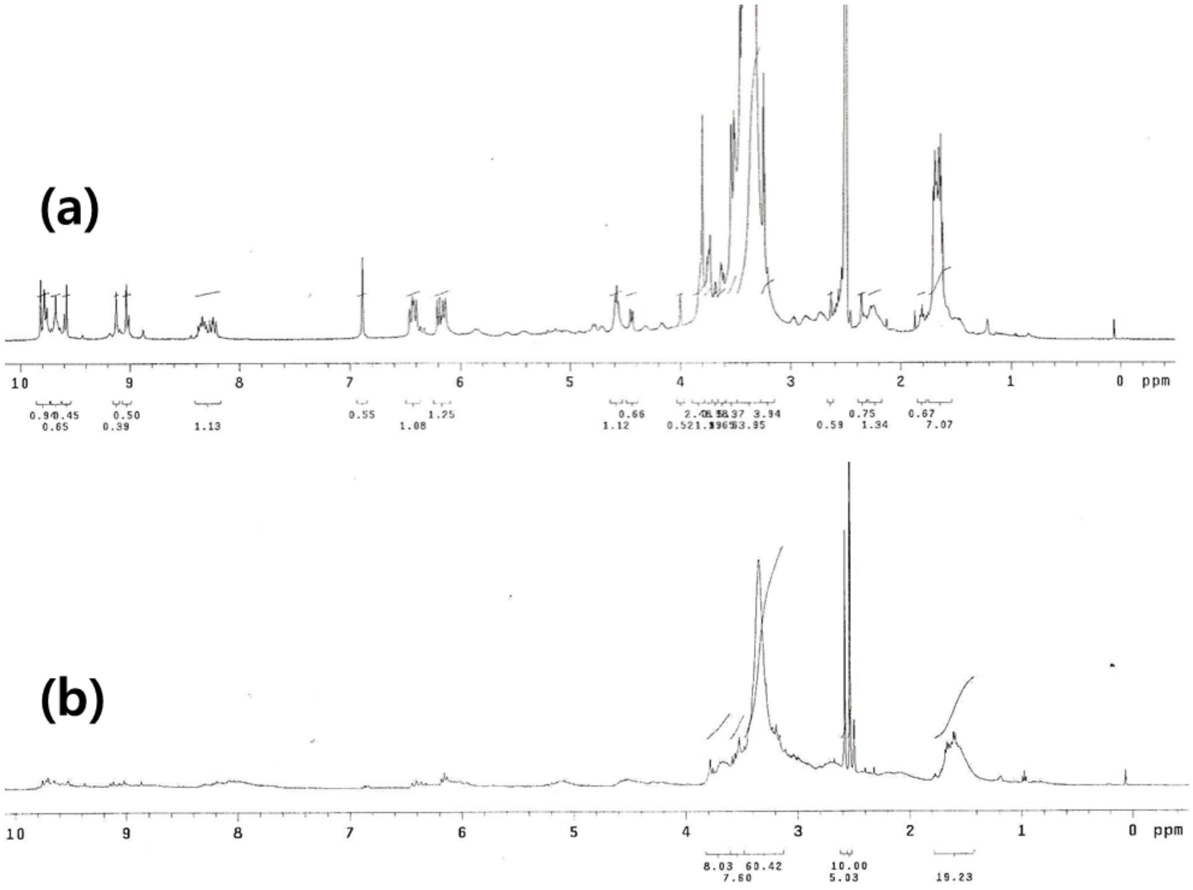

Figure S1. ^{1}H NMR spectra of Ce6 tetramer (a) and Ce6 decamer (b). For measurement of ^{1}H NMR (500 mHz) spectra, Ce6 tetramer and Ce6 decamer were dissolved in deuterated DMSO (DMSO d_6).

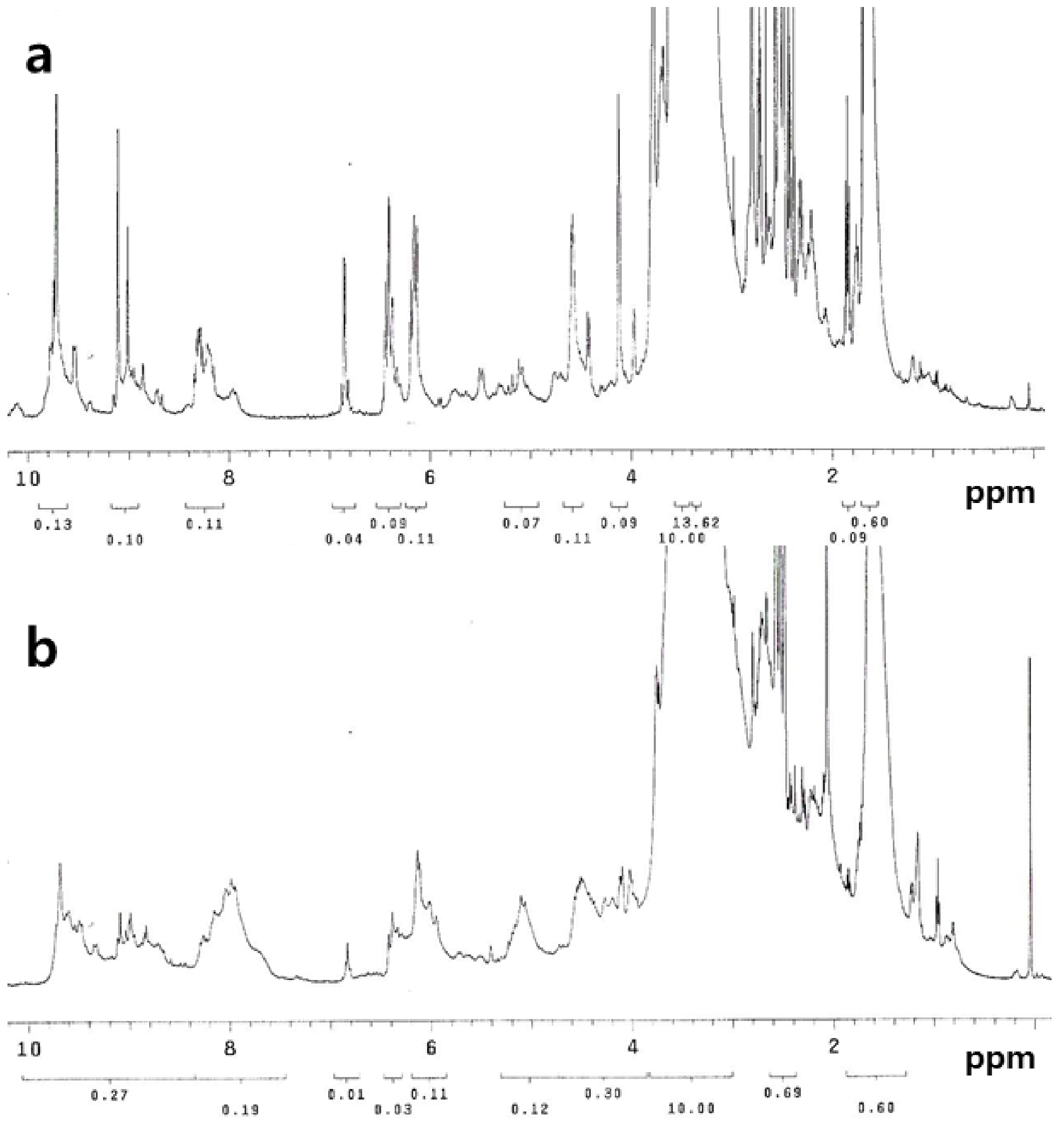

Figure S2. ^{1}H NMR spectra of Ce6 tetramer–MePEG 2K conjugates (a) and Ce6 decamer-MePEG 5K conjugates (b). For measurement of ^{1}H NMR (500 mHz) spectra, Ce6 tetramer–MePEG 2K conjugates and Ce6 decamer-MePEG 5K conjugates were dissolved in deuterated DMSO (DMSO d_6).

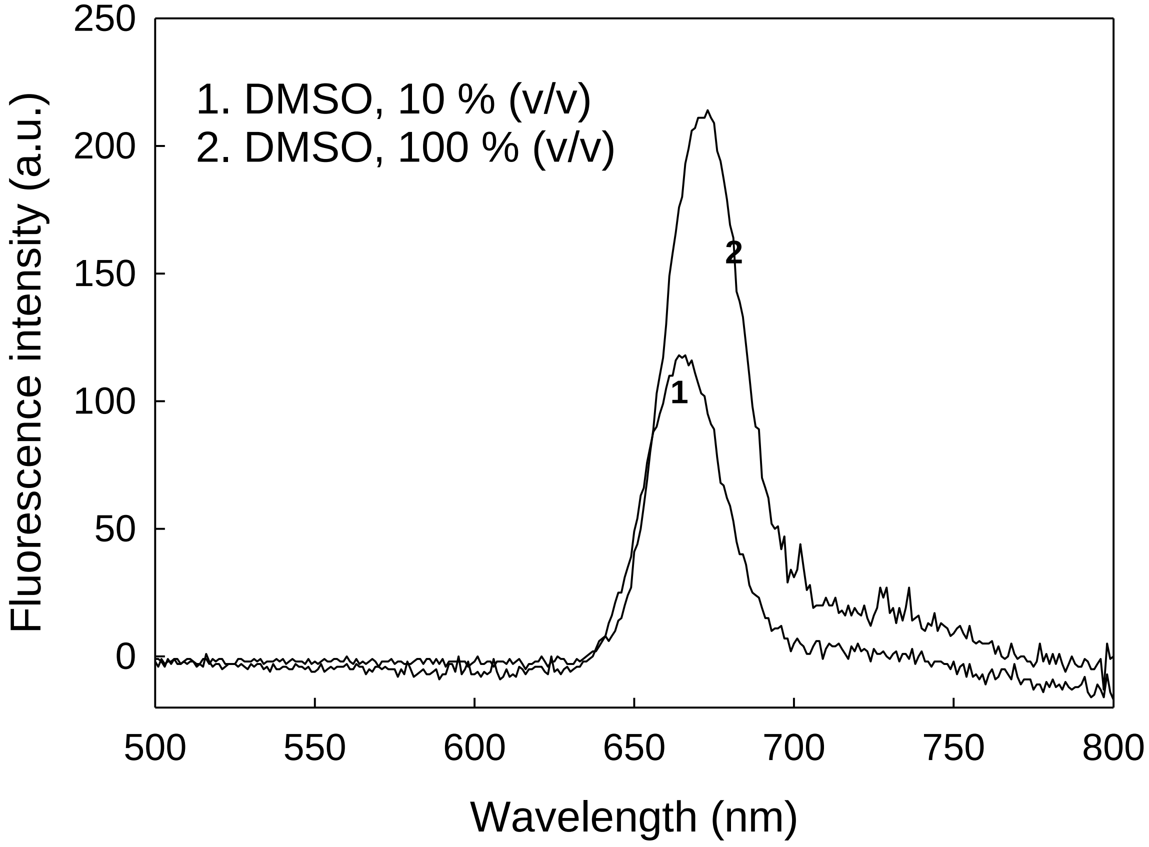

Figure S3. Fluorescence spectra of Ce6. Ce6 in DMSO was diluted with PBS (0.01M, pH 7.4) ten times for 10 % DMSO.

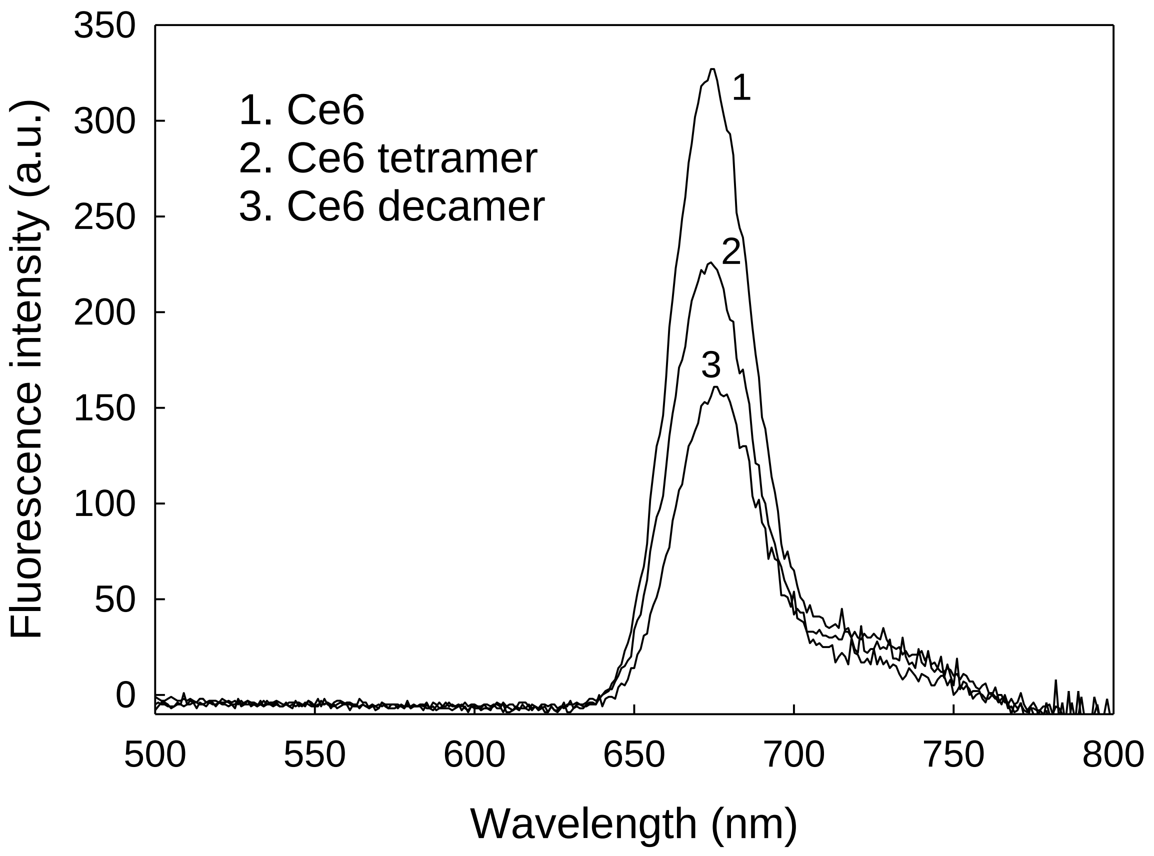

Figure S4. Fluorescence spectra of Ce6, Ce6 tetramer and Ce6 decamer. Ce6, Ce6 tetramer and Ce6 decamer was dissolved in DMSO (concentration: 0.1 mg/ml).
